# Infectious Disease Response in Tanzania: Enhanced Strategies From the 2023 to 2025 Marburg Outbreaks

**DOI:** 10.1002/puh2.70266

**Published:** 2026-04-30

**Authors:** Huda Haque, Faraan O. Rahim, Sameer Abbasi, Nam Nguyen, Erick Mahatara, Alison Jang, Ntuli A. Kapologwe

**Affiliations:** ^1^ Duke Global Health Institute Durham North Carolina USA; ^2^ Harvard Medical School Boston Massachusetts USA; ^3^ University of Illinois Urbana‐Champaign Champaign Illinois USA; ^4^ University of California San Diego San Diego California USA; ^5^ School of Medicine KCMC University Moshi Tanzania; ^6^ East, Central and Southern Africa–Health Community (ECSA–HC) Arusha Tanzania

**Keywords:** Africa, global health, Tanzania

## Abstract

The 2023 Marburg virus disease (MVD) outbreak in Tanzania, which resulted in nine confirmed cases and six deaths (case fatality rate [CFR]: 66.7%), served as a critical stress test of the national health system, revealing significant gaps in outbreak preparedness across surveillance, laboratory capacity, and infection prevention and control (IPC). This narrative review examines how Tanzania's public health response evolved between the 2023 and subsequent 2025 outbreak and assesses how response strategies changed between the two outbreaks. The 2025 outbreak was limited to two confirmed cases with no further transmission, a marked contrast to the 2023 outbreak. Between the two outbreaks, Tanzania implemented several critical interventions, including expansions of PCR diagnostic capacity, integration of electronic tracing modules, and public health communication strategies. This review synthesizes publicly available reports from the World Health Organization (WHO), Africa Centers for Disease Control and Prevention (Africa CDC), the Tanzanian Ministry of Health, and peer‐reviewed literature, and analysis focuses on three major response domains: infectious disease surveillance, IPC, and public health education as key factors associated with Tanzania's progress. The rapid containment of the 2025 outbreak suggests meaningful progress in Tanzania's public health capabilities and the need for continued, sustained investment in integrated surveillance systems and IPC infrastructure to maintain resilience against future outbreaks. Tanzania's adaptation and multi‐level interventions between 2023 and 2025 provide a model for regional health security in Africa.

## 1 Introduction

Marburg virus disease (MVD) is a severe hemorrhagic fever similar to Ebola, with a case fatality rate (CFR) as high as 88% [1]. Most MVD outbreaks have occurred in sub‐Saharan Africa, underscoring the importance of addressing the disease as a global health issue. In March of 2023, the Bukoba district of Kagera, Tanzania, reported its first confirmed outbreak of MVD, which resulted in nine confirmed cases and six deaths, with a CFR of 66.7% [2]. The 2023 outbreak strained the health system, revealing critical gaps in infectious disease management, including delayed surveillance and reporting, limited laboratory capacity, weak community engagement, and shortcomings in infection prevention and control (IPC) measures.

Less than 2 years later, Tanzania experienced a second MVD outbreak. On January 20, 2025, the United Republic of Tanzania declared an outbreak of MVD. During a national press conference that day, President Samia Suluhu Hassan confirmed the detection of the first case in the Biharamulo district of Kagera, located in the northwestern region of Tanzania. By February 10, the Ministry of Health had reported one additional confirmed case and eight probable cases linked to MVD [3].

In contrast to the 2023 outbreak, Tanzania responded swiftly in 2025. Immediately after the first case was declared, the Ministry of Health coordinated with the World Health Organization (WHO) to implement disease surveillance, testing, infection control, and cross‐border prevention measures [4, 5, 6, 7]. The government also secured $3 million from the WHO's contingency fund to support containment efforts, working alongside response teams from the Africa Centers for Disease Control and Prevention (Africa CDC). These coordinated efforts, along with effective IPC measures, were associated with the rapid containment of the outbreak [8]. No additional cases were confirmed beyond the initial two, and all 281 identified contacts completed their 21‐day monitoring period without developing symptoms [3].

This review aims to compare the 2023 and 2025 MVD outbreaks in Tanzania to identify and analyze key health system strategies that contributed to improved outbreak containment.

## 2 Methods

This narrative review examined Tanzania's 2023 and 2025 MVD outbreaks using publicly available sources. A targeted search was conducted across PubMed, WHO Disease Outbreak News reports, Africa CDC materials, CDC web materials, Tanzanian Ministry of Health documents, and other official public health websites between January 2025 and February 2025. Search terms included combinations of “Marburg,” “Marburg virus disease,” “Tanzania,” “Kagera,” “outbreak response,” “surveillance,” “infection prevention and control,” “IPC,” “contact tracing,” and “public health education.”

Sources were eligible for inclusion if they described the 2023 or 2025 Tanzania MVD outbreaks or discussed response strategies relevant to surveillance, IPC, or public health education. We included peer‐reviewed literature, official situation reports, ministry documents, outbreak updates, and organizational response summaries. We excluded duplicate records, sources not focused on Tanzania, and sources that did not provide substantive information on outbreak response. Because of the recency of the 2025 outbreak, gray literature from official public health organizations was included alongside peer‐reviewed publications.

Relevant sources were selected for inclusion based on their relevance to the 2023 and 2025 Tanzania MVD outbreaks and to infectious disease surveillance, IPC, and public health education.

### 2.1 Infectious Disease Surveillance

Limited surveillance capacity hindered early detection efforts during Tanzania's 2023 MVD outbreak. Primary healthcare centers, the first point of contact for affected patients, lacked rapid laboratory testing capabilities, leading to delays in case identification [9]. Ineffective surveillance and gaps in IPC protocols were further highlighted when two healthcare workers contracted MVD in primary healthcare centers [9]. Additionally, significant delays in case confirmation were observed, including an index case that was only identified 20 days after the patient's death. Surveillance tools such as Case Investigation Forms, contact listing forms, and follow‐up forms were also in short supply, further compromising response efforts [9].

Since then, Tanzania has strengthened its surveillance infrastructure [5, 8]. Expanded access to PCR diagnostic testing and genomic sequencing capacity in high‐risk regions such as Kagera improved the ability to rapidly confirm suspected cases [8]. The Africa CDC has provided technical assistance to enhance pathogen detection and genomic analysis. Additionally, collaboration between the Tanzania Red Cross Society and UNICEF significantly improved contact tracing efforts, ensuring the successful monitoring of 281 MVD contacts [3]. Although not yet confirmed due to funding uncertainties, USAID pledged $500,000 in late 2024 to bolster diagnostic capacity and expand contact tracing within primary healthcare centers [10].

To further enhance infectious disease surveillance, the Africa CDC has integrated an electronic tracing module into Tanzania's national disease surveillance system [11]. This system links local health data with national databases, improving the tracking of MVD and other infectious diseases. Electronic surveillance enables faster contact tracing and real‐time alert reporting, strengthening Tanzania's ability to respond swiftly to future outbreaks.

These improvements in surveillance were likely associated with earlier case detection and reduced delays in outbreak response by enabling faster laboratory confirmation and more efficient identification of transmission chains. The electronic tracing module, in particular, addressed possible structural bottlenecks by linking local health data to national databases in real time and reducing the risk of contacts being lost to follow‐up [11]. Thus, these changes and the associated improved outcomes underscore the importance of supporting diagnostic capacity and digital infrastructure to contain outbreaks. However, the sustainability of these gains depends on consistent funding for infrastructure maintenance and trained personnel, as well as internet connectivity in rural areas.

### 2.2 Infection Prevention and Control

During the 2023 outbreak, gaps in IPC contributed to increased MVD transmission. Screening and isolation centers established in high‐risk areas faced operational challenges. The risk of further spread was heightened as some patients left without completing treatment [9, 12]. Additionally, two healthcare workers were infected at primary healthcare centers [9]. Moreover, due to the limited accessibility of these centers, many rural areas lacked essential IPC resources [9]. Concerns were raised about the effectiveness of screening methods, as infected individuals may not exhibit symptoms until days after exposure [12].

Significant improvements have since been made to strengthen IPC during the current MVD outbreak in Tanzania. In collaboration with the WHO and USAID, Tanzania has established an infectious disease isolation unit in coordination with the Ministry of Health. This facility serves as a dedicated space for isolating MVD patients, providing immediate case management, including essential medications and supportive treatments [7]. Additionally, this unit, located at a one‐stop border point, enhances screening measures for travelers to prevent cross‐border transmission, particularly between Tanzania and neighboring Uganda. Furthermore, Africa CDC deployed a team of public health experts to work alongside community health workers (CHWs) in Kagera, Tanzania, to deliver immediate care such as medications, hydration, and oxygen management for patients, as well as personal protective equipment for healthcare personnel [8].

Efforts to further enhance IPC in Tanzania include a plan to recruit more than 130,000 community healthcare workers over the next 3 years, improving medical intervention and adherence to disease control protocols [13, 14]. International funding has also been mobilized to expand training for CHWs, ensuring better compliance with IPC protocols and broader access to basic healthcare services [11]. In December 2023, Gavi, the Vaccine Alliance, launched the African Vaccine Manufacturing Accelerator, committing $1 billion to support vaccine development and production in Africa [15]. Additionally, the Tanzanian Ministry of Health introduced a strategic plan extending to 2026 to promote research and laboratory expansion [16]. This initiative has led to the establishment of mobile laboratories and additional isolation centers in high‐risk regions, further strengthening the country's IPC capacity.

These IPC improvements were likely associated with reduced transmission among healthcare professionals, strengthened case management capacity during the 2025 outbreak, and may better equip Tanzania for future outbreaks, while also providing lessons for the broader region. The co‐location of this unit at a border crossing also reflects a more sophisticated understanding of transmission risk in mobile populations. However, several challenges warrant attention. The planned recruitment of over 130,000 CHWs over 3 years is ambitious and will require sustained funding [17]. This rapid expansion of the workforce will likely require substantial investment in training programs. Thus, future funding initiatives must continue to prioritize IPC efforts, ensuring the efficient integration of CHWs and the long‐term sustainability of interventions. Additionally, long‐term IPC resilience will require a transition from donor‐dependent to domestically financed infrastructure.

### 2.3 Public Health Education

Despite various response efforts, weaknesses in Tanzania's public health education system during the 2023 MVD outbreak may have contributed to the virus's spread. Stigmatization of survivors hindered disease control efforts, although limited public awareness about MVD—particularly regarding risk factors and protective measures—led to delays in case reporting and poor adherence to preventive guidelines [11, 12].

Since then, Tanzania has strengthened public health awareness through key partnerships. To combat stigma, the government launched the Marburg Survivors’ Program in collaboration with WHO, promoting the awareness of MVD and facilitating door‐to‐door outreach campaigns [18]. Additionally, outbreak preparedness was reinforced through the distribution of 14,220 IPC checklists to healthcare providers and the WHO‐led training of 35 IPC professionals [19].

An additional component of the 2025 response was the Ministry of Health's social media campaign, which provided MVD‐related education in Swahili, tailored to the Tanzanian context [14]. The campaign informed the public about the virus, high‐risk regions, symptoms, and preventive measures. It successfully promoted practices such as avoiding direct contact with infected individuals and bodily fluids, refraining from handling the bodies of MVD victims, ensuring a proper use of personal protective equipment among healthcare workers, and maintaining good hygiene, including frequent handwashing with soap and water. Leveraging digital platforms for public health education will be essential in future outbreak preparedness efforts.

Ultimately, these public health education initiatives were likely complementary to surveillance and IPC in the service of containing the 2025 MVD outbreak by increasing community awareness and improving adherence to preventive care. By tailoring messaging to the linguistic and cultural context of the affected population, the campaign improved accessibility in a way that generic health communications often fail to achieve. Additionally, the Marburg Survivors’ Program addressed stigma and thus may have encouraged care‐seeking behaviors. However, digital campaigns reach only those with internet access, which remains uneven across rural Tanzania, and future efforts should consider complementary CHW‐led education. It is imperative for future funding to continue prioritizing this aspect of outbreak control across Tanzania where it appears to have been valuable and in the broader region to strengthen trust and communication between communities and health systems and lead to more coordinated outbreak responses.

## 3 Discussion

The improved containment of the 2025 outbreak reflects the combined effects of strengthened surveillance, expanded IPC protocols, and public health strategies. Specifically, increased coordination between national and international public health partners and expanded diagnostic capacity likely contributed to reduced delays in case confirmation and containment. Additionally, the recent integration of electronic surveillance systems was associated with enhanced reporting efficiency and improved monitoring of exposed individuals, and public education campaigns bolstered outbreak containment efforts by connecting with individuals and rapidly disseminating critical information. Table 1 summarizes the key differences in response capacity and outcomes between the 2023 and 2025 outbreaks across the three domains examined.

**TABLE 1:** Comparison of MVD outbreak response strategies and outcomes, Tanzania 2023 versus 2025.

	2023 MVD Response	2025 MVD Response
**Surveillance**	Primary healthcare facilities and rural areas lacked rapid testing Delays in case confirmation Inadequate supply of contact‐listing/investigation forms	Increased access to PCR tests/genomic sequencing in rural areas Collaborations with Africa CDC, Red Cross, UNICEF to facilitate contact tracing USAID pledge to improve diagnostics New Africa CDC electronic tracing module
**Infection prevention and control**	Lack of adherence to isolation protocol Rural areas lacking prevention resources Inefficiency of screening methods in high‐traffic areas	Infectious disease isolation facility established by Ministry of Health Enhanced screenings at borders CHWs and public health workers deployed to provide PPE and medication Increased funding for R&D
**Public health education**	Significant stigma associated with MVD Lack of public awareness about risk factors/protective measures	Establishment of outreach program to combat MVD stigma Distribution of information to HCWs WHO‐led training of HCWs Social media public awareness campaign

*Note:* This table summarizes major differences in Tanzania's Marburg virus disease response between the 2023 and 2025 outbreaks across surveillance, infection prevention and control, and public health education.

Abbreviations: CDC, Centers for Disease Control and Prevention; MVD, Marburg virus disease; USAID, United States Agency for International Development; WHO, World Health Organization.

These domains were also closely connected in practice. Expanded surveillance capacity is most useful when paired with adequate IPC infrastructure and case management systems, whereas public health education can strengthen both surveillance and IPC by improving trust, encouraging earlier care‐seeking, reducing stigma, and supporting adherence to prevention guidance. Tanzania's progress may therefore reflect not only improvements within each domain, but also stronger coordination across them.

However, despite these improvements to the Tanzanian public health response, several challenges remain. The planned recruitment of more than 130,000 CHWs, along with overall sustained benefits of improved IPC, surveillance, and public health education campaigns, will require significant and continuous training and funding for this expansion to successfully integrate into existing health systems.

Tanzania's experience with MVD can also be situated within a larger regional context of improved public health efforts. For example, Uganda's response to Ebola outbreaks reflects improved contact‐tracing mechanisms and the integration of digital tools [20]. These regional parallels suggest that investments in the aforementioned three sectors can help support critical outbreak responses in the region [21]. Additionally, these findings are consistent with the International Health Regulations (IHR) core capacities framework, which emphasizes early detection, integrated surveillance systems, workforce development, and coordinated response mechanisms as key elements of outbreak preparedness [22].

Several limitations of this review should be acknowledged. As a narrative review relying on publicly available sources on a relatively recent outbreak, peer‐reviewed literature was largely unavailable, necessitating a heavier reliance on gray literature and media sources. Additionally, this review identifies associations between specific interventions and improved outcomes; it cannot establish causation. These limitations should be considered when interpreting the findings and their transferability to other settings.

## 4 Conclusion

Tanzania's response to the 2025 MVD outbreak demonstrated significant progress in infectious disease preparedness, building on lessons from the 2023 outbreak. Strengthened surveillance, improved IPC measures, and enhanced public health education were associated with the swift containment of the virus. Sustained investment from both international and national health partners in these areas will be essential to further enhance the country's outbreak preparedness and response.

## Author Contributions

**Huda Haque**: conceptualization, investigation, writing the original draft, and review and editing. **Faraan O. Rahim**: conceptualization, investigation, writing the original draft, and review and editing. **Sameer Abbasi**: investigation, writing the original draft, review and editing. **Nam Nguyen**: investigation, writing the original draft, review and editing. **Alison Jang**: investigation, writing the original draft, review and editing. **Erick Mahatara**: investigation and writing – review and editing. **Ntuli A. Kapologwe**: supervision, conceptualization, and writing – review and editing. All authors read and approved the final manuscript.

## Funding

The authors have nothing to report.

## Consent

The authors have nothing to report.

## Conflicts of Interest

The authors declare no conflicts of interest.

## Data Availability

The data that support the findings of this review article are derived from publicly available sources, including official reports from the World Health Organization (WHO), Africa Centers for Disease Control and Prevention (Africa CDC), and the United States Agency for International Development (USAID), as well as published news media and peer‐reviewed literature. All sources are fully cited in the References section of this manuscript. No new primary data were generated by the authors for this study.
